# Antibacterial Effects of Recombinant Endolysins in Disinfecting Medical Equipment: A Pilot Study

**DOI:** 10.3389/fmicb.2021.773640

**Published:** 2022-03-02

**Authors:** Yoon-Jung Choi, Shukho Kim, Sohyun Bae, Yoonjung Kim, Hyun-Ha Chang, Jungmin Kim

**Affiliations:** ^1^Department of Microbiology, School of Medicine, Kyungpook National University, Daegu, South Korea; ^2^Department of Allergy and Infectious Diseases, Kyungpook National University Hospital, Daegu, South Korea

**Keywords:** nosocomial infection, catheter, endolysin, drug resistance, disinfectant

## Abstract

Nosocomial infections caused by multidrug-resistant (MDR) bacteria are severe life-threatening factors. Endolysins (lysins) degrade the bacterial cell wall peptidoglycan and may help control pathogens, especially MDR bacteria prevalent in hospital settings. This study was conducted to verify the potential of lysin as disinfectant to kill bacteria contaminating medical devices that cause hospital infections. Eight catheters removed from hospitalized patients were collected and tested for their ability to kill bacteria contaminating the catheters using two lysins, LysSS and CHAP-161. Catheter-contaminating bacterial species were isolated and identified by 16s rRNA sequencing. From the eight catheters, bacteria were cultured from seven catheters, and five bacterial species (*Bacillus megaterium, Bacillus muralis, Corynebacterium striatum, Enterococcus faecium*, and *Staphylococcus epidermidis*) were identified. LysSS could inhibit catheter-contaminating bacteria, including *C. striatum* and *S. epidermidis*, compared with untreated controls but could not inhibit the growth of *E. faecium.* CHAP-161 showed more bactericidal effects than LysSS, but could not inhibit the growth of *S. epidermidis.* This study showed the potential of lysin as an alternative disinfectant for hazardous chemical disinfectants used in hospitals.

## Introduction

Healthcare-associated infection (HAI) is a major life-threatening factor in developed countries, and the importance of preventing HAI is increasing. In the modern medical environment, HAI is continuously increasing due to the development of anticancer therapy, increase in the number of patients with weakened immune function due to immunosuppressing drug use, generalization of invasive procedures, and increase in antimicrobial-resistant bacterial strains (Haque et al., 2018; Liu and Dickter, 2020; Monegro et al., 2021).

HAI is defined as an infection that occurs 48 h after a patient is admitted to a medical institution, within 2 weeks after discharge, or within 30 d after surgery (Monegro et al., 2021). HAIs include endogenous and exogenous infections; endogenous infection is caused by the overgrowth of microorganisms residing in the patient’s mouth and intestines due to a decrease in the patient’s immunity (Liu and Dickter, 2020; Monegro et al., 2021). In contrast, exogenous infection is caused by spreading through the environment of medical institutions, medical equipment, or people, such as medical personnel, hospital staff, and visitors. Common HAIs include central line-associated bloodstream infection (CLABSI), catheter-associated urinary tract infection (CAUTI), ventilator-associated pneumonia (VAP), and surgical site infection (SSI) (Mundy et al., 2000; Souto and Colombo, 2008; Vinodkumar et al., 2011; García-Solache and Rice, 2019; Liu and Dickter, 2020; Monegro et al., 2021; Souza et al., 2021). The major pathogens of HAI are *Staphylococcus aureus, Pseudomonas aeruginosa, Acinetobacter baumannii, Escherichia coli, Enterococcus* sp., and *Candida albicans*, and many clinical isolates exhibit multidrug resistance (Kollef et al., 1999; Stacy et al., 2021).

Research on endolysins (lysins) as a treatment for multidrug-resistant (MDR) bacteria is increasing (Marks and Sharp, 2000; Abedon et al., 2011; Young, 2013; Grishin et al., 2020; Orzechowska and Mohammed, 2021). Lysins are peptidoglycan hydrolyzing enzymes derived from bacteriophages that are expressed and worked inside the host bacteria in nature (Grishin et al., 2020). In the lytic cycle of bacteriophage, lysins digest the peptidoglycans of the bacterial cell wall and help the bacteriophage out from the host cell (Marks and Sharp, 2000; Abedon et al., 2011; Ul Haq et al., 2012; Young, 2013; Grishin et al., 2020). We recently published papers on recombinant lysin capable of lysing MDR bacteria (Kim et al., 2020a,b,c). Each lysin gene was cloned in an expression vector and expressed in an *Escherichia coli* strain with a six-histidine tag for easy purification. Recombinant LysSS protein developed from SS3e bacteriophage genome showed antibacterial activity against MDR *A. baumannii*, MDR *E. coli*, MDR *Klebsiella pneumoniae*, MDR *Pseudomonas aeruginosa, Salmonella* sp., and methicillin-resistant *S. aureus* (MRSA) (Kim et al., 2012, 2020c). Additionally, recombinant LysSAP26 protein developed from SAP26 bacteriophage genome inhibited the growth of carbapenem-resistant *A. baumannii*, carbapenem-resistant *E. coli*, carbapenem-resistant *K. pneumoniae*, carbapenem-resistant *P. aeruginosa*, MRSA, and vancomycin-resistant *E. faecium* (Rahman et al., 2011; Kim et al., 2020a). Therefore, we expect these lysins help control pathogens, especially MDR bacteria prevalent in hospital settings.

We conducted a pilot study to determine the potential of lysin as a disinfectant to kill bacteria contaminating medical devices that cause hospital infections. Eight catheters removed from hospitalized patients were collected and tested for their ability to kill bacteria contaminating the catheters using two lysins, LysSS and CHAP-161.

## Materials and Methods

### Bacterial Strains and Medium

The bacteria, including reference strains, were grown on blood agar plates (BAP, Gyeongin) at 37°C aerobically for 16–24 h. Mueller–Hinton agar (MHA, Difco), Mueller–Hinton broth (MHB, Difco), or both were used to determine the MICs and antibacterial activities. *Escherichia coli* DH5α and *Escherichia coli* BL21 (DE3) Star were cultured using Luria–Bertani broth (LB) with appropriate selective antibiotics for genetic works and lysin protein purification. LB with 15% glycerol was used to store bacterial stocks at −70°C.

### Isolation and Identification of Catheter-Contaminated Bacteria

Eight catheters removed from patients who visited Kyungpook National University Hospital (KNUH) were collected. Each catheter was placed in a 50-mL sterile plastic tube, and 10-mL saline (0.9% NaCl, JW Pharmaceuticals, Korea) was added and vortexed for 30 s. After the catheter was removed from the tube and the solution was centrifuged at 6,000 rpm for 5 min, the supernatant was discarded, and finally, 1 mL was left. The precipitate was completely resuspended, and 100 μL of the solution was spread on a BAP using a spreader, which was aerobically incubated at 37°C for 24–72 h. The bacterial colonies with different shapes and sizes were selected and isolated from the incubated BAP, which were subjected to 16s rRNA PCR and sequencing using 27F (5′- AGAGTTTGATCTGGCTCAG-3′) and 1492R (5′-CGGTTACCTTGTTACGACTT-3′) primers (Rosselli et al., 2016; Johnson et al., 2019).

### Preparation of Lysins

Two lysins, LysSS and CHAP-161, were used in this study. LysSS is an 18.5 kDa lysin consisting of 162 amino acids (Kim et al., 2020a,c). CHAP-161 is a 19.7 kDa protein consisting of 161 amino acids having a cysteine, histidine-dependent amidohydrolases/peptidases active domain made by deleting the amino acids at positions 162–251 of LysSAP26 lysin (Horgan et al., 2009; Rahman et al., 2011; Kim et al., 2020a). Those lysins were expressed successfully in an *E. coli* BL21 strain without any significant toxic effect to the BL21 strain. Each lysin has a six-histidine tag along with its C-terminal end and was purified using a His-trap column (GE Healthcare, Chicago, IL, United States) installed on a fast protein liquid chromatography (FPLC) AKTA Prime PLUS chromatography system (Pharmacia, United States) as described in our previous studies (Walmagh et al., 2012; Kim et al., 2020a,b,c). Residual imidazole in purified fractions was removed by dialysis with the lysis buffer. SDS-PAGE and Western blotting confirmed the purity and the size of both lysins. To detect His-tagging proteins by Western blotting, anti-His-Tag monoclonal antibody (Ab Frontier, Korea), and horseradish peroxidase (HRP)-conjugated polyclonal rabbit anti-mouse immunoglobulin G (Dako, Denmark) were used as primary and secondary antibodies, respectively. The concentration of purified protein was determined using the Pierce BCA Protein Assay Kit (Thermo Fisher Scientific, Waltham, MA, United States).

### Antimicrobial Activities of Lysins Against Catheter-Contaminating Bacteria

The antimicrobial activities of lysins were determined as the broth microdilution method for antibacterial susceptibility test with some modification (CLSI, 2020). Catheter solution (1 × 10^2^ CFU/50 μL) and 100-μL lysin were added to the wells of a 96-well microplate containing 50-μL 4 × MHB. The final LysSS and CHAP-161 lysin concentrations ranged from 1 to 200 μg/mL and 1–100 μg/mL, respectively. The mixture of catheter solution and lysin was incubated at 37°C for 48 h without agitation. During incubation, the optical density of the mixture was measured at 24, 30, and 48 h periods using a VersaMax microplate reader (Molecular Devices, San Jose, CA, United States) at 600 nm wavelength.

MICs of lysins against respective bacterial species isolated from catheters were determined using the modified broth microdilution method like described above. A total of 9 isolates (2 isolates of *Corynebacterium striatum*; CS1 and CS2, 4 isolates of *Enterococcus faecium*; EF1∼EF4, and 3 isolates of *Staphylococcus epidermidis*; SE1∼SE3) were used in this study. Fifty microliters of each bacterial solution (5 × 10^4^ CFU) and 100 μL of lysin (0–200 μg/mL, final concentration) were added to 50-μL 4 × MHB in a 96-well round-bottom microplate. Then, it was incubated at 37°C for 24 h, and bacterial growth at the bottom of each well was observed with the naked eye. Time-kill assay was performed with 1 × MICs of lysins against each bacterial species, which was described in Supplementary Materials.

To count viable bacterial cells after lysin treatment, 500-μL catheter solution (1 × 10^3^ CFU) and 1,000-μL lysin (0∼200 μg/mL) were added to 500-μL 4 × MHB in a 5-mL tube and incubated at 37°C for 48 h using an orbital shaker at 170 rpm. A hundred microliters of the mixture was taken twice at 24, 30, and 48 h periods, and it was subjected to viable cell counting with MHA plates. This experiment was performed three times independently.

### Statistical Analysis

The obtained data were analyzed by two-way ANOVA (McHugh, 2011; Van Hecke, 2013) with Tuckey *post hoc* tests, *p*-values lower than 0.05 were considered statistically significant (Murdoch et al., 2012). All data are presented as mean from minimum six replicates with standard deviation.

## Results

### Isolation and Identification of Bacteria From Catheters

As shown in Table 1, seven of eight catheters were contaminated with bacteria, and five bacterial species were identified. The identified bacterial species were *Bacillus megaterium, Bacillus muralis, Corynebacterium striatum, Enterococcus faecium*, and *Staphylococcus epidermidis*. Single bacterial species were isolated from three catheters (1-5655, 1-5656, and 1-5662), and two bacterial species were isolated from the other three catheters (1-5652, 3-5655, and 3-5652-A). Three bacterial species (*B*. *megaterium, C. striatum*, and *E. faecium*) were isolated from one catheter (2-5652). Additionally, *E. faecium* was isolated from four of eight catheters, and *S. epidermidis* and *C. striatum*, were isolated from three catheters. The number of bacteria ranged from 1 × 10^2^ to 1.1 × 10^4^ CFU/mL (Table 1).

**TABLE 1:** Identification of contaminated bacterial species isolated from the catheters.

Catheter number	Number of bacteria (CFU/mL)	Contaminating bacterial species
1-5652	3.00 × 10^3^	*Enterococcus faecium*, *Staphylococcus epidermidis*
1-5655	1.00 × 10^2^	*Staphylococcus epidermidis*
1-5656	1.00 × 10^2^	*Corynebacterium striatum*
1-5662	1.00 × 10^2^	*Bacillus muralis*
2-5652	5.94 × 10^3^	*Bacillus megaterium*, *Enterococcus faecium*, *Corynebacterium striatum*
3-5655	1.10 × 10^4^	*Enterococcus faecium*, *Corynebacterium striatum*
3-5652-A	2.17 × 10^3^	*Enterococcus faecium*, *Staphylococcus epidermidis*
3-5652-B	ND	ND

### Antibacterial Efficacy of Lysins Against Bacteria Isolated From Catheters

The antibacterial efficacy of two lysins (LysSS and CHAP-161) against three major bacterial species (*C. striatum, E. faecium*, and *S. epidermidis*) was evaluated by measuring their Minimum inhibitory concentrations (MICs) (Table 2). For quality control and comparison, *Acinetobacter baumannii* ATCC 17978 and *S. aureus* ATCC 25923 were included. The MIC values of LysSS against *A. baumannii* ATCC 17978 and *S. aureus* ATCC 25923 were 150 and 200 μg/mL, respectively, and those of CHAP-161 were 10 and 25 μg/mL, respectively (Table 2). The MIC values of LysSS against *C. striatum, E. faecium*, and *S. epidermidis* were 150, > 200, and 150 μg/mL, respectively. The MIC values of CHAP-161 against *C. striatum, E. faecium*, and *S. epidermidis* were 50, 25∼100, and > 100 μg/mL, respectively.

**TABLE 2:** Minimum inhibitory concentrations (MICs) of LysSS and CHAP-161 against representative bacterial strains.

Bacterium	Strain	Representative isolate designation	MIC (μg/mL)	
			LysSS	CHAP- 161
*Acinetobacter baumannii*	ATCC17978	–	150	25
*Staphylococcus aureus*	ATCC 25923	–	200	50
*Corynebacterium striatum*	Catheter isolate	CS1	150	50
		CS2	150	50
*Enterococcus faecium*	Catheter isolate	EF1	>200	50
		EF2	>200	50
		EF3	>200	100
		EF4	>200	50
*Staphylococcus epidermidis*	Catheter isolate	SE1	150	>100
		SE2	150	>100
		SE3	150	>100

Time-kill assays showed that LysSS and CHAP-161 were able to inhibit bacterial growth of all five strains tested compared to controls (Supplementary Figure 1). LysSS showed the strongest inhibitory effect on *A. baumannii* and *S. epidermidis*, a lower inhibitory effect on *S. aureus* and *C. striatum*, and the lowest inhibitory effect on *E. faecium*. The optical densities of *S. aureus* ATCC 25923 and *C. striatum* CS1 isolate were approximately doubled at 24 h periods, indicating that LysSS has not bactericidal effect against these bacteria. CHAP-161 could completely inhibit the growth of *A. baumannii* ATCC 17978, *S. aureus* ATCC 25923, *C. striatum* CS1, and *E. faecium* EF1, but not *S. epidermidis* SE1. However, the optical densities of LysSS-treated *E. faecium* and CHAP-161-treated *S. epidermidis* were lower than those of controls (32 and 78% inhibited, respectively) (Supplementary Figure 1).

### Antibacterial Effects of Lysins on Catheter-Contaminating Bacteria

**FIGURE 1:**
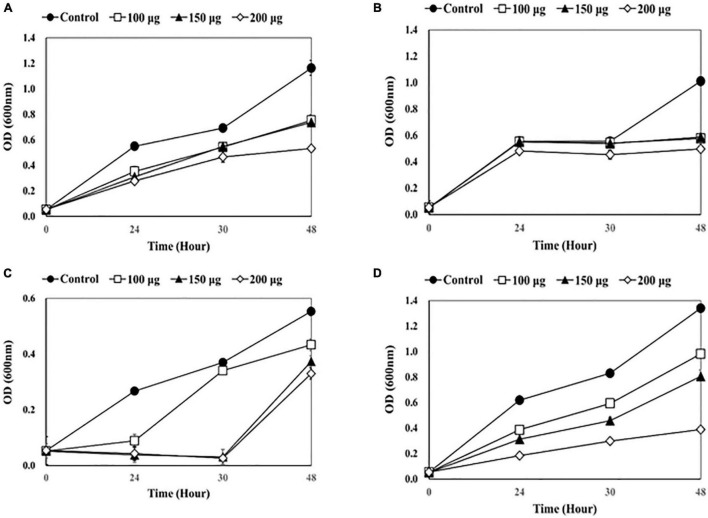
Measuring optical density (OD) after adding endolysin LysSS to catheter solution. **(A)** 1-5652, **(B)** 2-5652, **(C)** 3-5655, **(D)** 3-5652-A. These results were statistically evaluated and expressed with standard deviation error bars from three independent experiments.

**FIGURE 2:**
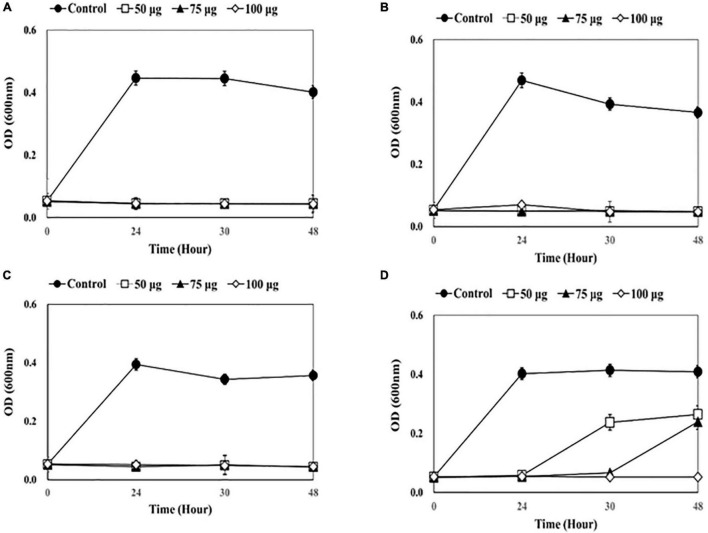
Measuring optical density (OD) after adding endolysin CHAP-161 to catheter solution. **(A)** 1-5652, **(B)** 2-5652, **(C)** 3-5655, **(D)** 3-5652-A. These results were statistically evaluated and expressed with standard deviation error bars from three independent experiments.

**FIGURE 3:**
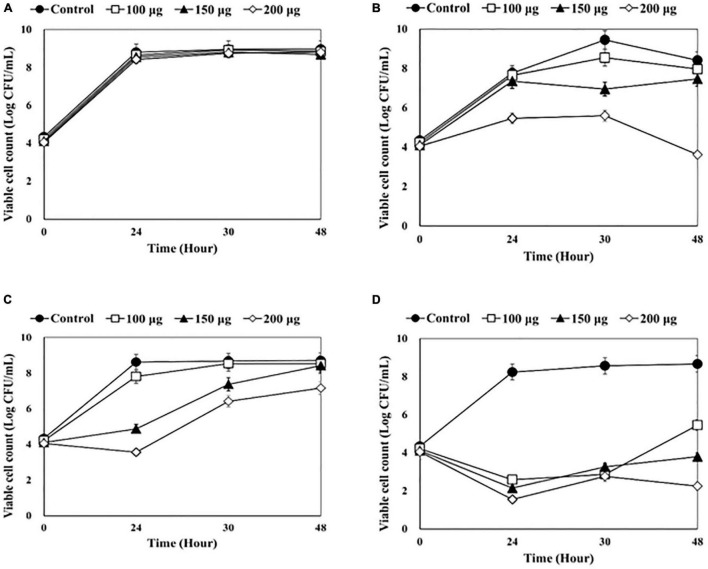
Counting viable bacterial cells after adding endolysin LysSS to the catheter solution. **(A)** 1-5652, **(B)** 2-5652, **(C)** 3-5655, **(D)** 3-5652-A. These results were statistically evaluated and expressed with standard deviation error bars from three independent experiments.

**FIGURE 4:**
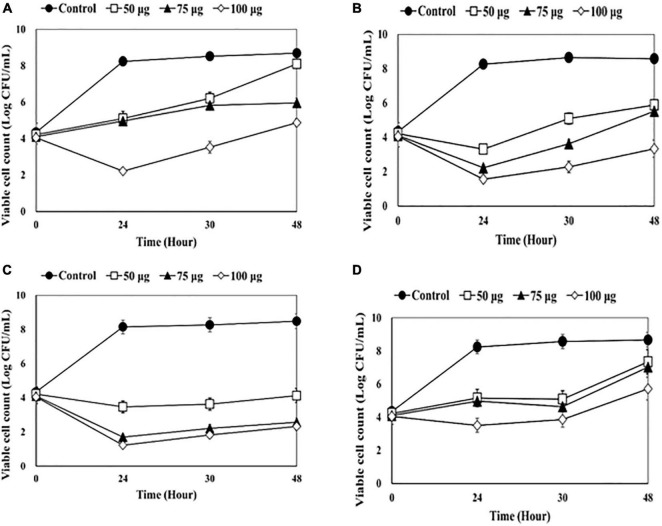
Counting viable bacterial cells after adding endolysin CHAP-161 to the catheter solution. **(A)** 1-5652, **(B)** 2-5652, **(C)** 3-5655, **(D)** 3-5652-A. These results were statistically evaluated and expressed with standard deviation error bars from three independent experiments.

The antibacterial effects of lysins as a disinfectant of medical equipment were evaluated using four catheters (1-5652, 2-5652, 3-5655, and 3-5652-A) contaminated with two or more bacterial species (Figures 1–4).

As shown in Figure 1, a dose-dependent reduction in bacterial growth was observed when LysSS was added. After treatment of catheter 1-5652 with LysSS at 100, 150, and 200 μg/mL, the optical density (OD) at 48 h showed decreases of 64.7% (0.753/1.163), 63.4% (0.737/1.163), and 45.8% (0.533/1.163) compared with the untreated control (Figure 1A). Likewise, the OD of catheter 2-5652 showed decreases of 78.3% (0.433/0.553), 67.5% (0.373/0.553), and 59.7% (0.330/0.553) (Figure 1B), Furthermore, the OD of catheter 3-5655 showed decreases of 57.1% (0.577/1.010), 58.2% (0.588/1.010), and 49.2% (0.497/1.010) (Figure 1C), and the OD of catheter 3-5652-A showed decreases of 73.2% (0.981/1.340), 60.1% (0.805/1.340), and 29.0% (0.389/1.340) (Figure 1D).

A dose-dependent reduction in bacterial growth was observed when CHAP-161 was added, which showed a greater decrease than LysSS (Figure 2). Bacterial growth of the three catheter solutions (1-5652, 2-5652, and 3-5655) was completely inhibited at 48 h, even when CHAP-161 was treated with the lowest concentration of 50 μg/mL in LysSS treatment (Figures 2A–C). The OD of catheter 3-5652-A at 48 h showed decreases of 64.8% (0.265/0.409), 58.7% (0.240/0.409), and 12.9% (0.053/0.409) at 50, 75, and 100 μg/mL of CHAP-161, respectively, compared with the untreated control (Figure 2D).

After LysSS treatment, the number of viable bacterial cells in the three catheters, except for catheter 1-5652 was significantly reduced compared with the control (Figure 3). The CFU of catheter 2-5652 at 100, 150, and 200 μg/mL of LysSS at 48 h showed reductions in log 0.17, log 0.29, and log 1.54 (Figure 3B). Likewise, CFU of catheter 3-5655 at 100, 150, and 200 μg/mL of LysSS at 48 h showed reductions in log 0.45, log 0.95, and log 4.8 (Figure 3C), and that of catheter 3-5652-A showed reductions in log 3.21, log 4.87, and log 6.42 (Figure 3D).

After treatment with CHAP-161 at a concentration of 50, 75, and 100 μg/mL, the number of viable bacteria at 24, 36, and 48 h was counted (Figure 4). The CFU of catheter 1-5652 at 50, 75, and 100 μg/mL at 48 h showed reductions in log 0.59, log 2.73, and log 3.82, respectively (Figure 4A). Likewise, that of catheter 2-5652 at 48 h showed reductions in log 4.35, log 5.91, and log 6.15 (Figure 4B); catheter 3-5655 at 48 h showed reductions in log 2.69, log 3.06, and log 5.24 (Figure 4C) and CFU of catheter 3-5652-A showed reductions in log 1.32, log 1.64, and log 2.95 (Figure 4D). The antibacterial effects (at 48 h post treatment) of LysSS and CHAP-161 on catheter-contaminating bacteria in four catheters (1-5652, 2-5652, 3-5655, and 3-5652-A) are summarized in Table 3.

**TABLE 3:** Antibacterial effects of LysSS and CHAP-161 on catheter-contaminating bacteria from four catheters (1-5652, 2-5652, 3-5655, and 3-5652-A).

Endolysin	Endolysin concentration (μg/mL)	Optical density ratio (%) at 48 h comparing with untreated control				Reduction of colony forming unit (logCFU/mL) at 48 h comparing with untreated control			
		1-5652	2-5652	3-5655	3-5652-A	1-5652	2-5652	3-5655	3-5652-A
LysSS	100	64.7	78.3	57.1	73.2	NR^b^	0.17	0.45	3.21
	150	63.4	67.5	58.2	60.1	NR	0.29	0.95	4.89
	200	45.8	59.7	49.2	29.0	NR	1.54	4.8	6.42
CHAP-161	50	CR^a^	CR	CR	64.8	0.59	4.35	2.69	1.32
	75	CR	CR	CR	58.7	2.73	5.91	3.06	1.64
	100	CR	CR	CR	12.9	3.82	6.15	5.24	2.95

*^a^CR, Complete reduction.*

*^b^NR, No reduction.*

## Discussion

The major nosocomial infectious bacteria are *S. aureus, E. coli, P. aeruginosa*, and *Enterococci* (Haque et al., 2018; Leitão, 2020). These bacteria have acquired multidrug resistance and stick to surfaces of medical equipment (e.g., catheter) as biofilm, which is super resistant to conventional antibiotics and disinfectants (Jett et al., 1994; Li et al., 2018). Antibiotic treatment with phage therapy provides better control of undesirable bacteria, particularly those microorganisms exhibiting multi-resistant phenotypes, and reducing the emergence of antimicrobial-resistant bacterial strains (Ul Haq et al., 2012; Villa and Sieiro, 2020; Łobocka et al., 2021). Bacteriophage-encoded endolysins are enzymes that act by digesting the peptidoglycan of bacterial cell walls. The potential of these proteins for controlling bacterial infections and preventing the pathogenic colonization of mucosal membranes has been demonstrated previously (Marks and Sharp, 2000; Abedon et al., 2011; Ul Haq et al., 2012; Grishin et al., 2020).

In this study, we conducted a pilot study to determine the potential of endolysin as a disinfectant to kill bacteria contaminating medical devices that cause hospital infections with two kinds of endolysins (LysSS and CHAP-161). LysSS is a 18.5 kDa protein consisting of 162 amino acids derived from *Salmonella*-specific phage (SS3e) and has a lysozyme-like domain (Kim et al., 2012, 2020c). CHAP-161 lysin is a recombinant protein made to include only a cysteine, the histidine-dependent amidohydrolase/peptidase (CHAP) domain of LysSAP26 (Kim et al., 2020a,c). LysSAP26 is a 29.1 kDa recombinant protein encoded in the 26th open reading frame of the genome of a bacteriophage (SAP26 phage) specific for *S. aureus* and composed of a CHAP domain (amino acids 1–109) and an unknown function protein (amino acids 110–251). The mechanism of action of endolysin with CHAP domain is known to cleave the peptide linkage of the peptidoglycan chain composed of N-acetylmuramic acid and N-acetylglucosamine. The truncated LysSAP26, CHAP-161, is more stable than LysSAP26 and has a higher bacterial degradation capacity (data not shown).

From eight urinary catheter, five bacterial species and 26 bacterial strains were identified in eight catheters. *S. epidermidis, E. faecium*, and *C. striatum* were major contaminating pathogens. Also, *S. epidermidis* and *E. faecium* are well-known nosocomial pathogens; in contrast, *C. striatum* is an emerging bacteria in the clinical settings that infects the skin and respiratory tracts and cause sepsis (Heyrman et al., 2005; Salzer, 2018; Clarke et al., 2020; Ge et al., 2020). Furthermore, *Bacillus muralis* and *B. megaterium* were identified with a few colonies in our isolation process, which have not been reported as pathogenic bacteria but are ubiquitous in the environment and even clinical settings (Heyrman et al., 2005; Ge et al., 2020). The significance of these bacteria regarding hospital infection control should be investigated in future studies. The results of MICs of LysSS and CHAP-161 against a single bacterial species showed that it was as effective against *A. baumannii* and *S. aureus* in our previous reports (Kim et al., 2020a,c). LysSS has no antibacterial effect on *E. faecium*, and CHAP-161 has no antibacterial effect on *S. epidermidis*. Therefore, the combination of lysins could expand its use as disinfectants in hospitals. Additionally, these two lysins could inhibit *C. striatum*, which was a novel finding regarding the two lysins’ antibacterial spectra.

To investigate the antibacterial efficacy of lysins, we used the OD measurement and viable bacterial cell counting methods. There are some discrepancies between the results of the two methods, presumably due to the differences in the bacterial culture conditions used in the two methods; static culture using a microplate in the OD measurement method and shaking aeration using a larger tube in viable bacterial cell counting method. The antibacterial spectrum of LysSS had *A. baumannii, E. coli, K. pneumonia, P. aeruginosa, Salmonella* Enteritidis, and *S. aureus*, and its MICs against these bacteria ranged from 250 to 750 mg/mL (Kim et al., 2020c). However, the growth of *E. faecium*, a major catheter-contaminating pathogen in this study, could not be inhibited by LysSS. Therefore, the antibacterial effects against four catheter solutions were limited due to the presence of *E. faecium.* In contrast, CHAP-161 showed a more drastic growth inhibition than LysSS for the four catheter bacterial solutions. This is probably because CHAP-161 can completely inhibit the growth of *E. faecium*.

This paper is not a study on the treatment outcome of patient with catheter-related infections in actual clinical practice, but rather a study on the disinfecting ability of endolysin against clinically isolated bacteria colonized in the urinary catheter. These colonized bacteria actually cause urinary tract infections in immunocompromised patients or in some patients. Therefore, in the case of patients belonging to the high-risk group for hospital infection, it may be possible to try to remove colonized bacteria in advance through endolysin treatment, and a study on this possibility is needed in the future.

There are some limitations in evaluating the effectiveness of our lysins for use as an alternative disinfectant in a hospital setting. First, this work was a pilot study; therefore, only few catheters were used to represent hospital equipment. Also, we may have missed some anaerobic and fastidious organisms because we only performed aerobic culture methods. The other point was that bacterial biofilm on various surfaces should be included because biofilm has been a major barrier to eliminating nosocomial infections (Kim et al., 2010, 2020b; Nelson et al., 2012; Li et al., 2018; Grishin et al., 2020). Nevertheless, this study is the first to show that lysin can be a new disinfectant. Therefore, although this report is a pilot study, it is valuable as the first attempt to improve the microbial environment with lysins in medical devices.

## Data Availability Statement

The original contributions presented in the study are included in the article/Supplementary Material, further inquiries can be directed to the corresponding author/s.

## Ethics Statement

The studies involving human participants were reviewed and approved by the Institutional Review Board of Kyungpook National University Hospital. Written informed consent for participation was not required for this study in accordance with the national legislation and the institutional requirements.

## Author Contributions

H-HC and JK: conceptualization and supervision. SK, Y-JC, H-HC, and JK: methodology. SK and JK: validation, formal analysis, data curation, writing—review, and editing and visualization. SK, H-HC, and JK: investigation. SB, YK, H-HC, and JK: resources. Y-JC and SK: writing—original draft preparation. SK: project administration. JK: funding acquisition. All authors have read and agreed to the published version of the manuscript.

## Conflict of Interest

The authors declare that the research was conducted in the absence of any commercial or financial relationships that could be construed as a potential conflict of interest.

## Publisher’s Note

All claims expressed in this article are solely those of the authors and do not necessarily represent those of their affiliated organizations, or those of the publisher, the editors and the reviewers. Any product that may be evaluated in this article, or claim that may be made by its manufacturer, is not guaranteed or endorsed by the publisher.
