# Supplementary material for: Antibacterial Effects of Recombinant Endolysins in Disinfecting Medical Equipment: A Pilot Study
Source: Front Microbiol. 2022 Mar 2;12:773640. doi: 10.3389/fmicb.2021.773640 (PMC8924034; doi:10.3389/fmicb.2021.773640)
Supplement: Supplementary file 1 [file Data_Sheet_1.docx]

**Supplementary materials**

**Materials & Methods.**

**Time-kill assay.** Time-kill assay experiments were performed with some modification using a microplate reader (CLSI). Bacterial solution (1×10^5^ CFU/50 µL) and 100 µL of lysin (LysSS or CHAP-161) were added to the wells of a 96-well microplate containing 50 µL of 4× MHB. The final concentrations of lysins were 1× MICs of respective bacteria. Bacteria that their MICs could not be measured were treated with the highest concentration of lysin, respectively (*E. faecium* for LysSS and *S. epidermidis* for CHAP-161). The negative control was the same volume of the lysis buffer instead of the lysin solution. The mixtures in a microplate was incubated at 37°C for 24 h without agitation and the bacterial growth was measured at 2, 4, 6, 8, 12, and 24 h periods using a VersaMax microplate reader (Molecular Devices, San Jose, CA, USA) at 600 nm wavelength. These experiments were carried out three times independently with triplicate each time.

**References**.

National Committee for Clinical Laboratory Standards. Methods for determining bactericidal activity of antimicrobial agents; approved guideline. NCCLS document M26-A. National Committee for Clinical Laboratory Standards, Wayne, PA (1999).

**Supplementary Figure Legend**

Suppl. Figure 1. Time-kill assay with 1× MICs of LysSS or CHAP-161 against *Acinetobacter baumannii* ATCC 17978 (a), *Staphylococcus aureus* ATCC 25923 (b), *Corinebacterium striatum* CS1 isolate (c), *Enterococcus faecium* EF1 isolate (d), and *Staphylococcus epidermidis* SE1 isolate (e). This results were statistically evaluated and expressed with standard deviation error bars from three independent experiments.
