# Supplementary figures and images for: Antibacterial Effects of Recombinant Endolysins in Disinfecting Medical Equipment: A Pilot Study
Source: Front Microbiol. 2022 Mar 2;12:773640. doi: 10.3389/fmicb.2021.773640 (PMC8924034; doi:10.3389/fmicb.2021.773640)

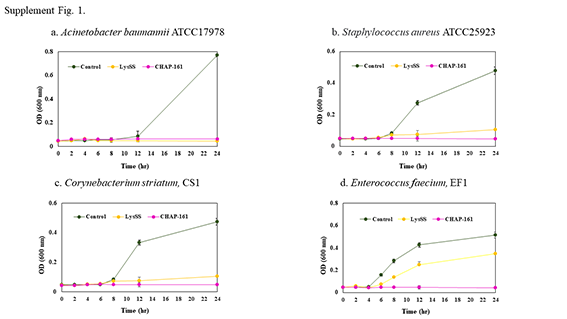

Supplement: Supplementary file 2 [file Image_1.TIF]

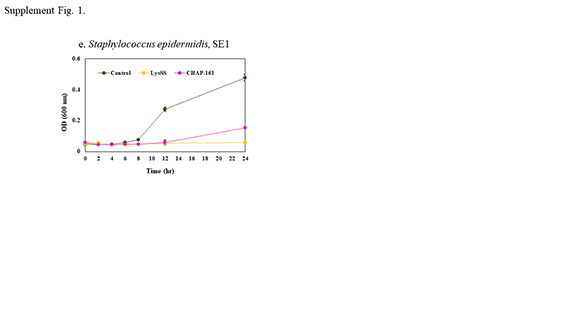

Supplement: Supplementary file 3 [file Image_2.TIF]
